# Die Genese des Informed Consent im Kontext der medizinischen Forschungsethik 1900–1931

**DOI:** 10.1007/s00120-023-02042-3

**Published:** 2023-02-21

**Authors:** Friedrich H. Moll, Matthis Krischel

**Affiliations:** 1 Institut für Geschichte, Theorie und Ethik der Medizin, Centre for Health and Society, Medizinische Fakultät, Heinrich-Heine-Universität, Düsseldorf, Deutschland; 2 Curator Museum, Bibliothek und Archiv, Deutsche Gesellschaft für Urologie e. V., Düsseldorf-Berlin, Deutschland; 3 Urologische Klinik, Urologischer Arbeitsplatz Krankenhaus Merheim, Kliniken der Stadt Köln gGmbH, Neufelder Straße 32, 51067 Köln, Deutschland

**Keywords:** Informierte Einwilligung, Menschenversuche, Albert Neisser, Medizinethik, Medizingeschichte, Human experimentation, Germany, Albert Neisser, Medical ethics, History of medicine

## Abstract

An der Wende zum 20. Jahrhundert rückte die Problematik des Humanexperimentes sowie die Notwendigkeit der Einwilligung hierzu in den Fokus der Mediziner und einer allgemeinen Öffentlichkeit. Es wird u. a. am Fall des Venerologen Albert Neisser die Entwicklung forschungsethischer Standards zwischen dem Ende des 19. Jahrhunderts und 1931 in Deutschland nachgezeichnet. Das aus der Forschungsethik stammende Konzept des Informed Consent ist heute auch in der klinischen Ethik von zentraler Bedeutung.

## Abstract (en)

At the turn of the 20th century, the problem of human experimentation and the need to obtain consent became more important among medical practitioners and the general public. The case of the venereologist Albert Neisser, among others, is used to trace the development of research ethics standards in Germany between the end of the 19th century and 1931. The concept of informed consent, which originated in research ethics, is also of central importance in clinical ethics today.

## Einführung

Mit der Implementierung des naturwissenschaftlichen Paradigmas in die Medizin zu Beginn des „langen 19. Jahrhunderts“ kamen auch ethische Problemstellungen vermehrt in den Fokus von Medizin und Gesellschaft, wie beispielsweise Fragen zur Forschung am Menschen [1], die zur Grundlage naturwissenschaftlicher Forschung wurden. Therapien wurden angewandt, von denen weder die Unschädlichkeit für die jeweiligen Patienten noch ihre Unschädlichkeit i. Allg. wissenschaftlich erwiesen waren [2].

In diesem besonders von Fortschrittsoptimismus geprägten Jahrhundert stellte sich für die forschenden Eliten kaum die moralische Frage nach der Zulässigkeit von Humanversuchen, insbesondere, wenn die Probanden aus vulnerablen Gruppen stammten und z. B. mittel- oder anderweitig rechtlos waren (Gefängnisinsassen, Arme, Menschen in den Kolonien, Kinder). Der Medizinhistoriker Richard Toellner (1930–2019) brachte das Dilemma für die Medizin auf den Punkt:

„Es ist unethisch, eine Therapie anzuwenden, deren Sicherheit und Wirksamkeit nicht wissenschaftlich geprüft ist; es ist aber auch unethisch, die Wirksamkeit einer Therapie wissenschaftlich zu prüfen.“ [3]

**Abb. 1:**
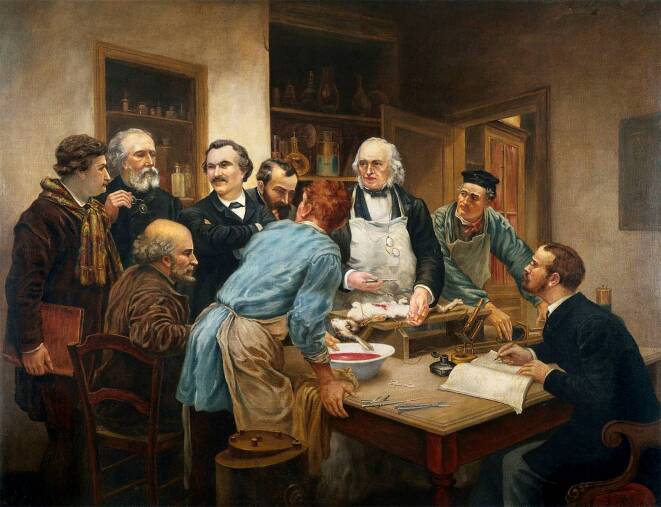
Claude Bernard 1813–1878 mit seinen Schülern (Vivisektion), Ölgemälde von Léon-Augustin Lhermitte (1844–1925), 1889, Académie Nationale de Médecine (Original mit Namenszug von Lhermitte links oben neben Wandregal), hier in der Version von Thomas Eakins (1844–1916). 86,5 × 112,5 cm. (Wellcome Collection no. 45530i. Mit freundl. Genehmigung)

Claude Bernard (1813–1878), Paris, der als Begründer der experimentellen Physiologie gilt ([4]; Abb. 1), stellte im Jahre 1865 in einer Publikation hierzu Regeln auf:

„Von den Versuchen, die man am Menschen ausführen kann, sind jene, die nur schaden können, verboten, jene, die harmlos sind, erlaubt, jene, die nützen können, geboten.“ [5]

Interessanterweise erfolgte die Übersetzung seines Werks ins Deutsche erst 1961 in einem Verlag der ehemaligen DDR.

Der Neurologe und Sexualmediziner Albert Moll (1862–1939), der ein frühes Werk zur Medizinethik im Jahre 1902 herausgab [6] und durch die zu dieser Zeit bestehenden Diskurse innerhalb der Medizin und der Öffentlichkeit hierzu angeregt worden war, schrieb in diesem Zusammenhang:

„Dient der Arzt ausschließlich dem Patienten, der sich ihm anvertraut hat, so ist die Ausnutzung des speziellen Krankheitsfalles für die Zwecke der wissenschaftlichen Forschung oft unmöglich; dient er aber ausschließlich der Lösung des wissenschaftlichen Problems, so gelangt er leicht dazu, das Wohl des Individuums, das sich ihm anvertraut hat, hintanzusetzen.“ [7, S. 215]

**Abb. 2:**
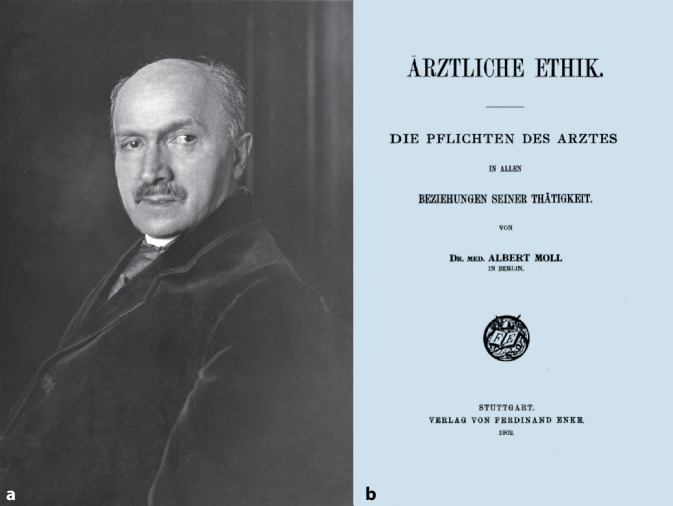
**a** Albert Moll (1862–1939) ca. 1930. **b** Frontispiz Ärztliche Ethik. (Enke Stuttgart 1902)

Damit bringt A. Moll eine zentrales Problem der wissenschaftlichen Medizin prägnant auf den Punkt, nämlich den Konflikt zwischen Heilkunde und Heilkunst (Abb. 2).

## Woher stammt das Konzept des Informed Consent?

In diesem Beitrag wird der Frage nachgegangen, welche Diskurse und Problemstellungen bereits im Jahre 1900 zu einer Anweisung des Preußischen Ministers der geistlichen‑, Kultus und Medizinalangelegenheiten führte, Ärzte zu verpflichten, Patienten aufzuklären und dies schriftlich festzuhalten. Die Entwicklung des Informed Consent – also der Einwilligung bzw. dem Einverständnis eines Patienten, nach adäquater Aufklärung entweder an Forschung teilzunehmen oder Diagnostik bzw. Therapie durchführen zu lassen – wird im deutschen Kontext bis ins Jahr 1931 verfolgt. Zum Schluss werden einige Beobachtungen zur Ausdehnung des Konzepts in die klinische Praxis und klinische Medizinethik erörtert.

Zwar hat Barbara Elkeles darauf hingewiesen, dass der Begriff des heutigen Informed Consent nicht passgenau auf die historische Situation angewandt werden könne, da die Auffassung von der Autonomie eines Patienten früher eine grundlegend andere war [8, S. 64, 9]. Gleichzeitig stecken in dem modernen Konzept viele historische Wurzeln, so dass ein historischer Blick auf das Thema durchaus das Potenzial birgt, die Praxis und den Wert der Patientenaufklärung im klinischen Kontext besser verständlich zu machen.

## Forschungsstand

Der Themenkomplex Informed Consent und Humanexperimente geriet ab den 1980er-Jahren in den Fokus der metaethischen Forschung, wobei das Konzept des Informed Consent auch historisiert wurde [10–16]. Für die historisch-ethische Analyse im deutschen Raum stehen dabei etwa die Arbeiten von Barbara Elkeles, Lutz Sauerteig und Matthis Krischel [8, 9, 17–19], Susan Ledererʼs Veröffentlichungen nehmen die USA und die internationale Dimension in den Blick [20, 21]. Hierbei werden in den letzten Jahren auch Ikonen der Medizingeschichte wie beispielsweise die Nobelpreisträger Robert Koch (1843–1910) und Paul Ehrlich (1854–1915) kritisch diskutiert und Fragen über die ethische Qualität ihrer Forschung gestellt [22–30].

Auch die Grenzen des Informed Consent selbst werden ausgelotet, etwa wenn Kritiker das Konzept für ein Produkt einer autonomiedominierten Medizinethik halten, die in der klinischen Praxis zu einer Unterbewertung und Vernachlässigung intersubjektiver Werte führt und die Tore zu wunscherfüllender Medizin öffnet [31, 32].

## Der „Fall Neisser“

In diesem Zusammenhang sind für die Urologie besonderes der Venerologe Albert Neisser und verschiedene venerologische Versuche am Menschen von besonderer Bedeutung. So warf man Neisser und anderen Forschern kurz vor 1900 vor, für ihre Untersuchungen über Harnröhrenentzündungen „mit Vorliebe jugendlichen Individuen (14–18 Jahre alt), die nie eine Gonorrhö durchgemacht hatten“, ohne deren Wissen Bakterienaufschwemmungen in die Harnröhre injiziert zu haben [33, 34, 87]. Der Fall sollte zu einer Beschäftigung der Öffentlichkeit und des preußischen Parlaments mit Fragen der medizinischen Forschungsethik führen.

**Abb. 3:**
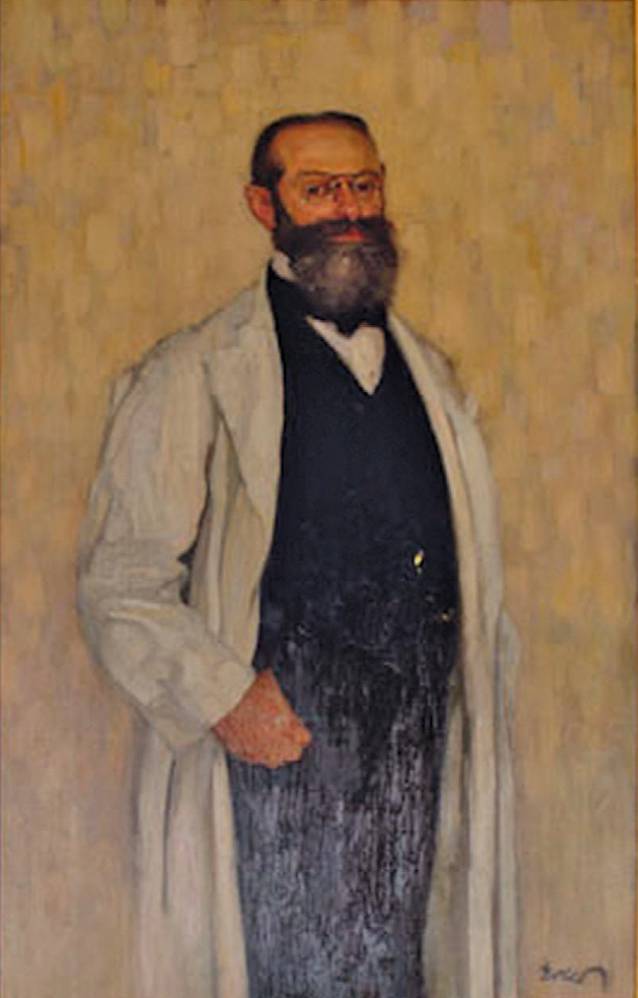
Albert Neisser1855–1916, Porträt Fritz Erler 1868–1940, zwischen 1900–1908 entstanden, ausgestellt Kunstausstellung Verein Berliner Künstler im Künstlerhaus Berlin, März 1908, Medizinische Fakultät Universität Breslau (Wroclaw). (Aus Scholz 2004 [38])

Geboren wurde Albert Neisser als Sohn eines jüdischen Arztes und Geheimen Sanitätsrats in Breslau, wo er auch die Schule besuchte. Hier legte er mit Paul Ehrlich im Jahre 1872 die Reifeprüfung ab. Im selben Jahr begann er an der Schlesischen Friedrich-Wilhelms-Universität zu Breslau das Studium der Humanmedizin. 1877 legte er das Staatsexamen ab und wurde mit einer Arbeit über die Bandwurmerkrankung promoviert. Im Jahre 1879 gelang Neisser die bahnbrechende Entdeckung des Erregers der Gonorrhö (Tripper), den er „Micrococcus“ nannte, von Paul Ehrlich später in „Gonococcus“ (Neisseria gonorrhoeae, Neisser-Diplokokken) umbenannt [35]. Nach seiner Habilitation im Jahre 1880 in Leipzig wurde Neisser zum Privatdozenten ernannt. 1882 übernahm er als a. o. Professor die Leitung der Dermatologischen Universitätsklinik in Breslau. 1907 wurde er zum o. Professor ernannt. Albert Neisser war Mitglied der Leopoldina ([36–39]; Abb. 3).

In den letzten Jahren des 19. Jahrhunderts hatte Paul Ehrlich in Berlin die Serumtherapie der Diphtherie entwickelt [17, 40, S. 143]. Dieses Prinzip versuchte Albert Neisser in Breslau auch auf die Behandlung der Syphilis zu übertragen, der Erreger war zu diesem Zeitpunkt noch nicht entdeckt. Denn erst 1905 entdeckten Fritz Schaudinn (1871–1906) sowie Erich Hoffmann (1868–1959) am Kaiserlichen Gesundheitsamt den Syphiliserreger (Spirochaeta pallida). Neisser fand bei diesen Forschungen ein gut vorbereitetes Feld, in dem sich staatliche Interessen der Forschungsförderung und die junge, aufstrebende medizinische Disziplin der Dermatologie im Labor trafen. Während sich der Staat mit dem Bau der Laboratorien im internationalen Kampf gegen die Seuche Syphilis profilieren wollte, fördert der Ort die Arbeit am disziplinären Profil des Wissenschaftlers und des aufstrebenden universitären Faches Dermatologie, welches (noch) nicht an allen deutschen Hochschulen vertreten war [15, S. 31].

**Abb. 4:**
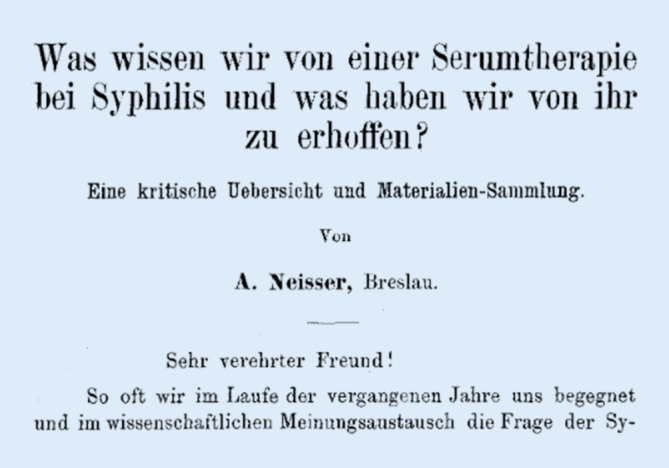
Ausriss Neisser: Was wissen wir von einer Serumtherapie bei Syphilis und was haben wir von ihr zu erhoffen? (Festschrift Josef Pick Archiv für Dermatologie und Syphilis 1898)

Hier entstand Neissers Schrift, welche später den Stein des Anstoßes ins Rollen bringen sollte ([41]; Abb. 4).

Mit Aussagen wie

„Es wäre übrigens sehr leicht gewesen, durch Centrifugiren oder durch geeignetes Filtriren sich noch eine grössere Sicherheit zu verschaffen“ [41, S. 485–486]

oder

„Die Möglichkeit, dass die intravenösen Infusionen geschadet haben könnten, kann also nicht geleugnet werden“ [41, S. 488]

dokumentierte Neisser seine eigene Fahrlässigkeit im Umgang mit den Versuchspersonen. Aus einigen seiner Aussagen spricht sogar eine gewisse Geringschätzigkeit:

„Ich selbst freilich bin davon, dass die genannten Prostituierten auf andere ‚normale‘ Weise inficirt worden sind, vollkommen überzeugt“ [41, S. 488].

In Folge von Neissers Veröffentlichung erhielt die Tageszeitung *Münchener Freie Presse* dann eine anonyme Zuschrift, in der über die Seruminjektionen Neissers berichtet wurde (*Münchener Freie Presse*, 20. Januar 1899). Der Artikel, der in der Reihe „Arme Leute in Krankenhäusern“ erschien, gab dann den Startschuss zu einer Debatte um die Experimente Albert Neissers [15, S. 83], die auf ein bloß subjektives, auf sehr schwachen Füßen stehendes Vermuten hin 8 gesunden Versuchspersonen das Syphilisserum subkutan injizierte. Vier von diesen menschlichen Versuchspersonen blieben während jahrelanger Beobachtung von Syphilis frei, die 4 anderen, von denen 3 mit intravenösen Infusionen „behandelt“ worden waren, erkrankten später an unverkennbar. Hieraus wurde dann, auch im juristischen Sinne der Tatbestand hergeleitet. Der zeitgenössische Pressediskurs wurde im Jahre 2007 von Katja Sabisch akribisch nachgezeichnet [15].

## „Wertlose Körper“ und der Beginn einer medizinethischen Debatte

Kritiker und Gegner der Schulmedizin – darunter Tierrechtler, Impfgegner und Naturheilkundler – machten bald aufmerksam auf die besonderen ethischen Fragen des Humanexperiments. Ihnen war bei der Durchsicht der medizinischen Fachliteratur aufgefallen, dass zahlreiche Forscher Humanexperimente vorgenommen hatten – nicht zu Heilzwecken, sondern lediglich zu wissenschaftlichen Forschungszwecken. Dabei waren in der Blütezeit der Bakteriologie meist mittellose und ungebildete Kranke mit Erregern von Syphilis, Scharlach, Erysipel oder Gonorrhö absichtlich infiziert worden. Oft wurden Patienten ausgewählt, denen – nach Ansicht der Forscher – kaum mehr geschadet werden konnte: Sterbende, für die sich unter Medizinern ein eigener Ausdruck durchgesetzt hatte: „Corpora vilia“ (wertlose oder billige Körper). Der Historiker und Friedensnobelpreisträger Ludwig Quidde (1858–1941), der den Diskurs um Neisser ab 1898 mit anstieß, übersetzte diese Bezeichnung einer vulnerablen Gruppe polemisch als:

„Schlechte wertlose Masse, an der man herumexperimentiert.“ [42, 43]

Auch der Eingangs bereits zitierte Berliner Neurologe und Sexualwissenschaftler Albert Moll (1862–1939), der im Jahre 1902 ein beachtetes frühes Werk zur Medizinethik verfasste hatte, äußerte in der Zeitschrift *Zukunft* kritisch:

„dass es unter dem ‚Stande‘, der jetzt laut nach staatlichem Schutz gegen Kurpfuscher schreit, der seine ‚eigenen‘ Angelegenheiten nach den Prinzipien seiner ‚eigenen‘ und ganz besonderen Ehre bei verschlossenen Thüren selbst regeln will, dass es unter diesem Stande Subjekte gibt, denen man nicht ein Kind zur Impfung anvertrauen darf, weil man nicht sicher ist, dass sie es nicht zu nichtswürdigen Experimenten mit Syphilis-Gift missbrauchen werden. . .Und wer weiß, ob eine solche Handlungsweise nach dem ‚Ehrenkodex‘ der ‚Kollegen‘ infam ist, oder nur ‚überwissenschaftlich‘!“ [7, S. 213]

A. Moll ging es jedoch eher um eine grundsätzliche Erörterung des Problems als um den konkreten „Fall Neisser“. Er fährt fort:

„Ebenso wäre es eine Vorbedingung für die Besserung der heutigen Zustände, dass der Kultusminister nicht einseitig Schritte gegen einen einzelnen Forscher einschlage, dessen Verhalten er missbilligt. Das würde den Eindruck der Parteilichkeit machen. Wenn der Minister Untersuchungen anstellt, dann soll er gegen alle Schuldigen vorgehen, nicht gegen den einen, übrigens sehr verdienten Mann, der gerade im Parlament angegriffen wurde.“ [7, S. 217]

**Abb. 5:**
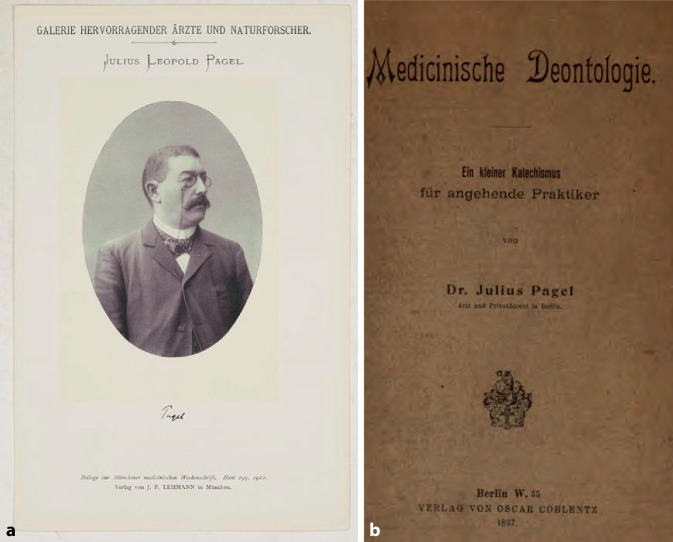
**a** Leopold Pagel, Medizinhistoriker, 1851–1912 Lehmanns Galerie hervorragender Ärzte und Naturforscher, Beilage *Münchener Medizinische Wochenschrift*. Lehmanns, 1912. **b** Buchcover Medizinische Deontologie 1897, Verlag Oscar Coblentz, Berlin

Der Berliner Medizinhistoriker Leopold Pagel (1851–1912; Abb. 5), der ebenfalls zu dieser Zeit mit einer medizinischen Deontologie in Erscheinung getreten war, äußert sich hingegen viel mehr im „Mainstream“ der Ärzteschaft. Seine ethische Auffassung war die einer uneingeschränkten Standessolidarität unter Ärzten und Wissenschaftlern:

„Für uns liegt die Ethik des Falles Neisser in der Mahnung, dass die Forschung nicht eher ruhen darf, als bis sie ein für das Wohl der Menschheit so ungeheuer wichtiges Problem, welches die Geister seit Jahrhunderten beschäftigt, definitiv verabschiedet ist. Verflucht ist die Wissenschaft, kann man mutatis mutandis mit dem Dichter sagen, die nicht alles setzt [auf] ihren Fortschritt! Wehe ihr, wenn sie durch äußere Schranken, welche ihr Alte Jungfer-Sentimentalität oder Laienkritik auferlegen will, sich vom Verfolg der Wahrheit abbringen lässt!“ [44]

Auch die Auswahl der Versuchspersonen durch Neisser nahm Pagel in Schutz:

„Ob man übrigens Puellae publicae als diejenigen ‚corpora vilia‘ ansehen darf, die in dem Spruch gemeint seien: ‚Fiat experimentum in corpore vili‘ bleibe hier unerörtert. Jedenfalls fällt als ein weiterer Milderungsgrund ins Gewicht, dass die von Neisser benutzten Personen sämtlich krank waren, und dass ein therapeutischer Effekt ganz gewiss a priori nicht ausgeschlossen schien.“ [45]

Die widerstreitenden Positionen innerhalb der Medizin und in der breiten Gesellschaft zur Ethik des Humanexperiments in den Jahren um 1900 zeigen, dass es bereits eine öffentliche Meinung gab, nach welcher Versuchspersonen vor Missbrauch zu schützen waren. Auch innerhalb der Ärzteschaft war dies keine randständige Position, wie die Aussage eines von Neissers Assistenten verdeutlicht, aus der durchaus Unrechtsbewusstsein spricht:

„Schließlich bemerke ich, dass wir an Personen, welche sich ausdrücklich weigerten, weder Aderlässe noch Injectionen ausgeführt haben. Der Anschuldige hatte uns im uebrigen instruirt, gegen Personen, die sich weigerten, von etwaigen Maßnahmen Abstand zu nehmen, er wusste aber wie ich annehme sehr wohl, dass wir, wenn wir ihnen die volle Wahrheit mittheilten, nicht zum Ziele gelangt wären.“ [46]

## Urteil gegen Neisser und die „Preußischen Anweisungen“

Am 29.12.1900 fällte der Königlichen Disziplinarhof für Nicht-richterliche Beamte sein Urteil. Neisser wurde zu einer (moderaten) Geldbuße von 300 Mark verurteilt und erhielt einen Verweis [47, 48]. Seiner Karriere bereitete der Skandal jedoch keinen Abbruch. Nur wenige Jahre später wurde er in Breslau zum ordentlichen Professor ernannt und als Neisser im Jahre 1916 infolge einer Sepsis nach Blasensteinlithotripsie starb, schien in den Nachrufen noch einmal das volle Verständnis seiner Kollegen für ihn und sein seinerzeitiges Handeln auf [49–51].

Mit dem Urteil gegen Neisser ließ der amtierenden Preußischen Minister der geistlichen‑, Kultus- und Medizinalangelegenheiten Konrad von Studt (1838–1921) jedoch mit den „Anweisung an die Vorsteher der Kliniken, Polikliniken und sonstigen Krankenanstalten“ einen ersten Kodex zur Forschung am Menschen ergehen. Darin wurde einleitend festgestellt:

„[Ich] weise […] darauf hin, dass medicinische Eingriffe zu anderen als diagnostischen, Heil- und Immunisierungszwecken, auch wenn die sonstigen Voraussetzungen für die rechtliche und sittliche Zulässigkeit vorliegen, doch unter allen Umständen ausgeschlossen sind, wenn es sich um eine Person handelt, die I) noch 1) minderjährig oder aus anderen Gründen nicht vollkommen geschäftsfähig ist; 2) die betreffende Person nicht ihre Zustimmung in unzweideutiger Weise erklärt hat; 3) dieser Erklärung nicht eine sachgemäße Belehrung über möglicherweise nachteilige Folgen vorausgegangen ist. II Zugleich bestimme ich, daß 1) Eingriffe dieser Art nur von dem Vorsteher selbst oder mit besonderer Ermächtigung desselben vorgenommen werden dürfen; 2) bei jedem derartigen Eingriffe die Erfüllung der Voraussetzungen zu I Nr 1‑3 und II Nr 1 sowie alle näheren Umstände des Falles auf dem Krankenblatte zu vermerken sind. III. Die bestehenden Bestimmungen über medizinische Eingriffe zu diagnostischen, Hei- und Immunisierungszwecken werden durch dies Anweisungen nicht berührt“ [9, S. 209, 52]

Die preußische Direktive von 1900 muss zweifellos als ein frühes Dokument gelten, welches einen Standard des Informed Consent in der medizinischen Forschung am Menschen einfordert. Es kam aber nicht auf Initiative der Ärzteschaft oder von Forschungsinstitutionen zustande, sondern war das politische Resultat eines breiten, öffentlichen Diskurses und der darin enthaltenen öffentlichen Kritik von Seiten der politischen Presse im Deutschen Reich sowie des Parlaments in Preußen am Missbrauch von Menschen bei wissenschaftlichen Experimenten [53, S. 19].

Doch leider – und dies ist nicht untypische für forschungsethische Codices – verhinderten die Preußischen Anweisungen in der Folge nicht, dass es zu weiteren Medizinskandalen kam. So ließ beispielsweise Paul Ehrlich den Wirkstoff Arsphenamin zur Syphilistherapie an mehreren 100 Patienten ausprobieren, ohne zuvor deren Einwilligung dazu eingeholt zu haben [40]. Im Jahre 1912 ließ der Berliner Tuberkuloseforscher Friedrich Franz Friedmann (1876–1953) 53 Waisen impfen, was eine Berichterstattung in der 1876 gegründeten sozialdemokratischen Zeitschrift *Vorwärts* auslöste, ohne vorher die Einwilligung der Erziehungsberechtigten noch die Zustimmung des zuständigen Dezernenten eingeholt zu haben [54].

## Die Richtlinien des Jahres 1931 für das Deutsche Reich

**Abb. 6:**
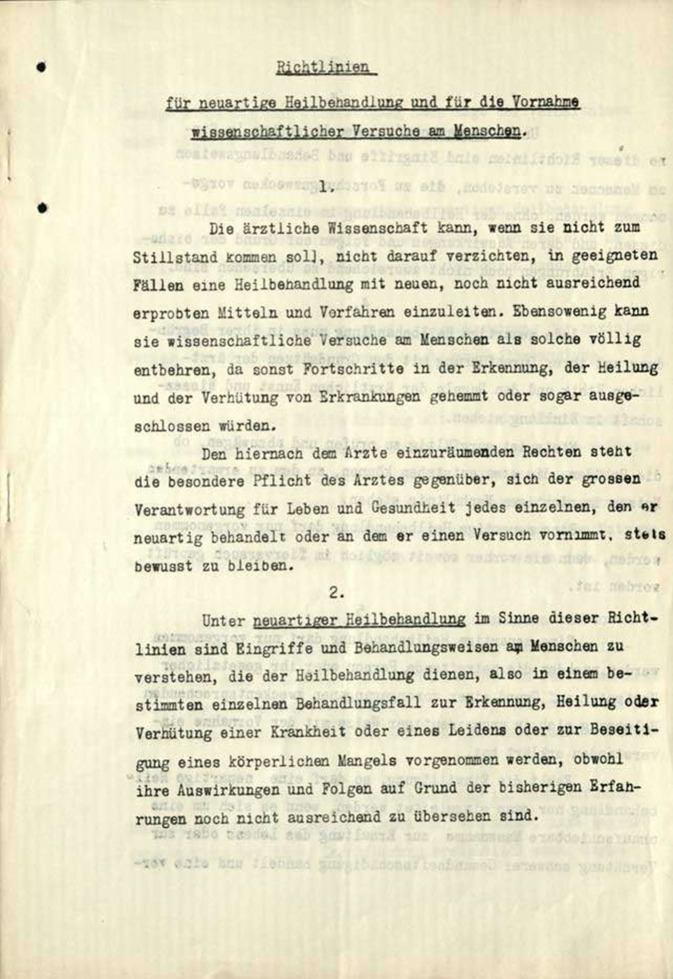
Richtlinien für neuartige Heilbehandlungen und für die Vornahme wissenschaftlicher Versuche am Menschen. (Staatsarchiv Freiburg, mit freundl. Genehmigung)

In den 1930er-Jahren wurde der Informed Consent in der klinischen Forschung in Deutschland in den „Richtlinien für neuartige Heilbehandlung und nur die Vornahme wissenschaftlicher Versuche am Menschen*“* vom 28.02.1931 in einem Rundschreiben des Reichsministers des Innern Joseph Wirth (1879–1956) erneut als Grundsatz der medizinischen Forschungsethik benannt. Diese Richtlinie behielt bis zum Jahre 1945, also während der gesamten NS-Zeit, formal Gültigkeit ([55–57]; Abb. 6).

Hintergrund war u. a. die Impfkatastrophe von Lübeck bei der Einführung der BCG-Impfung (Lebendimpfung mit dem Bacillus Calmette-Guérin [BCG] zum Schutz vor Tuberkulose) im Jahre 1931, bei der insgesamt 77 Menschen, überwiegend Kinder, zu Tode kamen. Hier war es in einem Labor zur Verunreinigung des Impfstoffs mit aktiven Tuberkuloseerregern gekommen, da weder eine eindeutige räumliche Trennung zwischen den Impfkulturen und den gleichzeitig verarbeiteten, infektiösen Tuberkulosekulturen noch notwendige Tierversuche zur Kontrolle des Impfstoffs erfolgt waren [58].

Darüber hinaus gab es in der Weimarer Republik weitere Versuche an Menschen, die einen breiten Pressediskurs hervorgerufen hatten [43].

Die Richtlinien stellten fest, dass zur Weiterentwicklung der ärztlichen Wissenschaft und Heilbehandlung wissenschaftliche Versuche am Menschen unverzichtbar wären. Hieraus erwüchse für den Arzt ein Recht zur Ausführung von Versuchen am Menschen einerseits, andererseits eine Pflicht und große Verantwortung für das Leben und die Gesundheit des einzelnen Versuchspatienten. Dabei wurde zwischen therapeutischen („neuartige Heilbehandlung“ Art. 2) und nicht-therapeutischen Versuchen („wissenschaftliche Versuche“ Art. 3) unterschieden und eine vorherige tierexperimentelle Testung sowie eine Schaden-Nutzen-Abwägung (Art. 4) vorgeschrieben. Analog dieser Differenzierung galten für wissenschaftliche Versuche strengere Durchführungsbestimmungen (Art. 12ff) als bei neuartigen Heilbehandlungen. Für beide waren eine fachgerechte wissenschaftliche Durchführung nach den „Regeln der ärztlichen Kunst und Wissenschaft“ (Art. 4) und eine Risiko-Nutzen-Analyse (Art. 4) erforderlich. Versuche am Menschen durften nur durchgeführt werden,

„nachdem die betreffende Person oder ihr gesetzlicher Vertreter auf Grund einer vorausgegangenen zweckentsprechenden Belehrung sich in unzweideutiger Weise mit der Vornahme einverstanden erklärt hat“ (Art. 5).

Während der Einwilligungsteil eindeutig festgelegt war, blieb ein Interpretationsspielraum darüber, was in der Praxis mit „zweckentsprechender Belehrung“ gemeint war. Der Begriff „Belehrung“ sprach noch die Sprache eines paternalistischen Arzt-Patient-Verhältnisses, in dem Arzt nicht nur ein medizinischer Wissensvorsprung, sondern darüber hinaus die moralische Autorität zur Belehrung des Patienten eingeräumt wurde. Da die Patientenaufklärung nach dem Ziel der Forschungsstudie „zweckentsprechend“ ausgerichtet sein sollte, muss kritisch gefragt werden, ob es sich dabei wirklich um eine angemessene patientenbezogene Aufklärung handelte. Auf der anderen Seite wurde dem Patienten bei der Einwilligung eine eigenständige Entscheidung eingeräumt [53, S. 20]. Weiterhin wurde eine schriftliche Dokumentationspflicht des Arztes über die wissenschaftliche Untersuchung selbst (Forschungsprotokoll) und über das erfolgte „Einverständnis“ des Kranken nach „zweckentsprechender Belehrung“ (heute als gültiger Informed Consent bezeichnet) gefordert (Art. 10).

Die Richtlinien von 1931 berücksichtigten sogar die sozialen und institutionellen Rahmenbedingungen, in denen Forschungsversuche stattfanden und ging damit sowohl über den Nürnberger Kodex von 1947als auch über die erste Fassung der Deklaration von Helsinki hinaus [59]. Bereits 1931 wurde die Ausnutzung einer sozialen Notlage für die Durchführung von medizinischer Forschung am Menschen verurteilt. Die Regelungen von 1931 enthielten bereits Elemente moderner Medizinethik wie Forschungsplan mit differenzierten Studienphasen (Tierexperiment vor Humanexperiment), Klärung der Verantwortung, Risiko-Nutzen-Analyse, Aufklärung und Einwilligung (Informed Consent) und Dokumentationspflicht sowie besondere Schutzbestimmungen für Minderjährige, sterbende Patienten und sozial Schwache (vulnerable Gruppen). Die grundlegende, medizinethische Differenzierung zwischen therapeutischer (Heilversuch) und nicht-therapeutischer (Humanexperiment) Forschung besitzt bis heute Gültigkeit. Dieser Differenzierung folgte die „Deklaration von Helsinki“ ([60]; 1964) des Weltärztebundes, die Rechtsprechung des Bundesgerichtshofes und das deutsche Arzneimittelgesetz [19, 53, S. 21, 55, 61].

## Nürnberger Kodex und Deklaration von Helsinki

Bekanntlich konnten aber auch die Richtlinien von 1931 die Medizinverbrechen im Nationalsozialismus, darunter die oftmals mörderischen Humanexperimente ohne Einwilligung der Versuchspersonen in den Konzentrationslagern, nicht verhindern [62]. Diese Menschenversuche wurden im Nürnberger Ärzteprozess angeklagt, mit den Urteilen wurde mit dem Nürnberger Kodex ein neues normatives Dokument veröffentlicht [63–65]. Die Formulierung dieses Kodex u. a. den Sorgen der American Medical Association und der British Medical Association geschuldet, dass das Bekanntwerden der deutschen Verbrechen das weltweite Vertrauen der Bevölkerung in die medizinische Forschung beeinträchtigen könnte [66, S. 3]. Der Nürnberger Kodex blieb aber in einigen Bereichen ein Kompromiss, der beispielsweise eine zunächst angedachte Forderung nach dem Schutz von psychisch kranken, nicht-einwilligungsfähigen Patienten am Ende nicht (mehr) enthielt [66, S. 288]. Entsprechend gering blieb sein Effekt auf die weltweite Forschung. Im Kontrast dazu steht, dass heute sogar manchmal eine Überregulation beklagt wird [67–70].

Seit 1964 regelt die vom Weltärztebund verabschiedete Deklaration von Helsinki die ethischen Grundsätze der Forschung am Menschen. Um mit den Entwicklungen in der Medizin und den Ansprüchen an Probanden- und Patientenschutz mitzuhalten wurde die Deklaration bis heute neun Mal überarbeitet, zuletzt 2013. Seit der ersten Revision 1975 enthält sie die Verpflichtung, Forschungsethikkommissionen zu konsultieren. Diese wurde sukzessive auch in deutsches und europäisches Recht umgesetzt [19].

## Forschungsethik in der Urologie und in chirurgischen Fächern

Auch für Pionieroperationen in der Urologie, beispielsweise die erste indizierte Nephrektomie an der Wäscherin Margarethe Kleb (1820–1877) 1869 [71] oder die erste totale Zystektomie an dem Kölner Schreiner Theodor Baum (1830–1887) im Jahre 1887 [72], lassen sich zwar medizinisch wissenschaftliche Begründungen und ausführliche Beschreibungen des Eingriffs in der zeitgenössischen Literatur finden, die sogar die sozialen Hintergründe der Patienten aufleuchten lassen [73]. Zur Einholung einer Patientenaufklärung wird in den Publikationen und Primärquellen keine Stellung genommen.

Bis heute gibt es Hinweise darauf, dass nicht bei allen chirurgischen Innovationsstudien die Ethikkommissionen a priori konsultiert und die Patienten über den Innovationscharakter des Eingriffs angemessen aufgeklärt werden [74]. Solches Verhalten lässt sich für die Ärzte seit der zu Beginn des 20. Jahrhunderts aus dem paternalistisch aufgefassten ärztlichen Selbstverständnis und ärztlich definierten Heilungsauftrag „Salus aegroti suprema lex“ („Das Heil des Kranken ist oberstes Gesetz“) ableiten. In operativen Fächern lässt sich aus der innewohnenden Charakteristik ein Studiendesign analog der Pharmaforschung mit verblindeten Therapiearmen oder Placebogruppen schwieriger umsetzen. Der Arzt allein hatte und hat zu entscheiden und sieht auch heute weitere Einrichtungen in diesem Prozess als hinderlich und die wissenschaftliche Freiheit bedrohend an [75, 76]. Zwar hat ein allgemeiner Wandel der Werteauffassung stattgefunden [77]. Man kann aber nur über aktuelle Fragen diskutieren, wenn man die historische Entwicklung berücksichtigt [78].

## Fazit für die Praxis

- Heute hat sich die Relevanz des Informed Consent von der Forschungsethik in die klinische Ethik und Praxis ausgeweitet. Dabei hat sich zum Thema eine reiche Publizistik entfaltet, in welche auch rechtliche Betrachtungen einfließen [79]. Die stellvertretende Entscheidung für nicht einwilligungsfähige Patienten oder eine Therapiezieländerung (i. d. R. fort von einer kurativen, hin zu einer palliativen Therapie) stellen konkrete Probleme der klinischen Praxis dar [80–82]. Allgemein wird zwischen therapeutischer Aufklärung und Diagnoseaufklärung unterschieden [83].
- Häufig orientiert sich die Medizinethik heute an den vier bioethischen Prinzipien von Tom Beauchamp und James Childress („Georgetown-Mantra“). Hier werden vier grundlegende ethische Prinzipien formuliert: Respekt vor dem Selbstbestimmungsrecht von Personen („respect for autonomy“), Wohltun („beneficence“), Nichtschaden („non-maleficence“) und Gerechtigkeit („justice“) [84]. Diese Prinzipien mittlerer Reichweite finden in vielen Bereichen der klinischen Medizin Anwendung [85]. Aus dem Respekt vor Patientenautonomie kann im Kontext der Forschungsethik auch die Pflicht abgeleitet werden, in der Forschung – ebenso wie in der klinischen Praxis – einen Informed Consent einzuholen, welcher gemeinsam mit einer Risiko-Nutzen-Analyse und einer gerechten Probandenauswahl die Basis ethisch fundierter Forschung am Menschen bilden kann [86].

## References

[1] Heinrichs B (2006). Forschung am Menschen Studien zu Wissenschaft und Ethik.

[2] Toellner R, Ders (1990). Problemgeschichte: Entstehung der Ethik-Kommissionen. Die Ethik-Kommission in der Medizin: Problemgeschichte, Aufgabenstellung, Arbeitsweise, Rechtsstellung und Organisationsformen Medizinischer Ethik-Kommissionen.

[3] Toellner, Toellner R (1990). Problemgeschichte: Entstehung der Ethik-Kommissionen. Die Ethik-Kommission in der Medizin. Problemgeschichte, Aufgabenstellung, Arbeitsweise, Rechtsstellung und Organisationsformen Medizinischer Ethik-Kommissionen.

[4] Buchholz G 1985 Die Medizintheorie Claude Bernards. Diss Med. Aachen, Murken-Altrogge, Herzogenrath.

[5] Bernhard C (1961). Einführung in das Studium der experimentellen Medizin (Paris 1865).

[6] Moll A (1902). Ärztliche Ethik: Die Pflichten des Arztes in allen Beziehungen seiner Thätigkeit.

[7] Moll A 1899 Versuche am lebenden Menschen. Zukunft 22. März S 215.

[8] Elkeles B (1989). Die schweigsame Welt von Arzt und Patient. Einwilligung und Aufklarung in der Arzt-Patient-Beziehung des 19. und frühen 20. Jahrhunderts. Med Ges Gesch.

[9] Elkeles B (1996). Der moralische Diskurs über das medizinische Menschenexperiment im 19. Jahrhundert.

[10] Faden RR, Beauchamp TL (1986). A history and theory of informed consent.

[11] Kevorkian J (1985). A brief history of experimentation on condemned and executed humans. JAMA.

[12] von Engelhardt D, Labisch A, Spree R (1989). Entwicklung der ärztlichen Ethik im 19. Jahrhundertmedizinische Motivation und gesellschaftliche Legitimation. Medizinische Deutungsmacht im sozialen Wandel.

[13] Ajlouni KM (1995). History of informed medical consent. Lancet.

[14] Vollmann J, Winau R, Baron JH (1996). History of informed medical consent Comment. Lancet.

[15] Sabisch K (2007). Das Weib als Versuchsperson Medizinische Menschenexperimente im 19. Jahrhundert am Beispiel der Syphilisforschung.

[16] Schmidt U, Frewer A, Sprumont D (2020). Ethical research: the declaration of Helsinki, and the past, present, and future of human experimentation.

[17] Elkeles B (1985). Medizinische Menschenversuche gegen Ende des 19. Jahrhunderts und der Fall Neisser. Medizinhist J.

[18] Sauerteig L (2000). Ethische Richtlinien, Patientenrechte und ärztliches Verhalten bei der Arzneimittelerprobung (1892–1931). Medhist J.

[19] Krischel M (2021). The institutionalization of research ethics committees in Germany—international integration or in the shadow of Nuremberg?. Eur J Hist Med Health.

[20] Lederer S (1995). Subject to science. Human experimentation before the second world war.

[21] Lederer S, Frewer A (2007). Forschung ohne Grenzen. Die Ursprünge der Deklaration von Helsinki. Standards der Forschung. Historische Entwicklung und ethische Grundlagen klinischer Studien.

[22] Mildenberger F (2013). Kein Heil durch Arsen? Die Salvarsandebatte und ihre Konsequenzen. Fachprosaforsch Grenzüberschreitungen.

[23] Zimmerer J (2020) Der berühmte Forscher und die Menschenexperimente. https://www.spiegel.de/geschichte/robert-koch-der-beruehmte-forscher-und-die-menschenexperimente-in-afrika-a-769a5772-5d02-4367-8de0-928320063b0a. Zugegriffen: 31. Apr. 2021

[24] Bauche M (2006) Robert Koch, die Schlafkrankheit und Menschenexperimente im kolonialen Ostafrika. http://www.freiburg-postkolonial.de/Sei-ten/robertkoch.htm. Zugegriffen: 30. Apr. 2021

[25] Eckart WU (2002). The colony as laboratory: German sleeping sickness campaigns in German East Africa and in Togo, 1900–1914. Hist Philos Life Sci.

[26] Hüntelmann AC, Gaudilliere P, Hess V (2013). Making Salvarsan: experimental therapy and the development and marketing of Salvarsan at the crossroads of science, clinical medicine, industry, and public health. Ways of regulation drugs in the 19 th and 20th century.

[27] Noack Th 2002 Eingriffe in das Selbstbestimmungsrecht des Patienten. Juristische Entscheidungen, Rechtspolitik und ärztliche Positionen 1890–1960. Med. Diss. Berlin.

[28] Schirren C (2001). Versuche am Menschen in der Dermatologie vor 100 Jahren und heute. Dermatologe.

[29] Roelcke V, Maio G (2004). Twentieth century ethics of human subjects research. Historical perspectives on values, practices, and regulations.

[30] Schmidt U, Frewer A (2007). History and theory of human experimentation. The declaration of Helsinki and modern medical ethics.

[31] Vollmann J (2000) Ethische Probleme des informed Consent Konzeptes. In: Vollmann J (Hrsg) Aufklärung und Einwilligung in der Psychiatrie. Monographien aus dem Gesamtgebiete der Psychiatrie, Bd. 96. Steinkopff, Heidelberg, S 99–132 10.1007/978-3-642-53783-7_4

[32] Vollmann J (2000). Ethische Probleme des Informed Consent-Konzeptes. Aufklärung und Einwilligung in der Psychiatrie.

[33] Tashiro E (1991). Die Waage der Venus. Venerologische Versuche am Menschen zwischen Fortschritt und Moral.

[34] Grosz S, Kraus R (1898). Bacteriologische Studien über den Gonococcus. Arch Dermatol Syph.

[35] Schadewaldt H, Coulston Gillispie C (1974). Neisser, Albert Ludwig Sigesmund. Dictionary of scientific biography.

[36] Schmitz S (1968). Albert Neisser Leben und Werk auf Grund neuer, unveröffentlichter Quellen.

[37] Bendick C, Scholz A (2005). Albert Neissers Expeditionen nach Java 1905 und 1907. Hautarzt.

[38] Scholz A, Plewig G, Kaudewitz P, Sander CA (2004). Albert Neisser und Fritz Erler: Brücken zwischen Breslau und München. Fortschritte der praktischen Dermatologie und Venerologie.

[39] Barth J, Löser Ch (2019). Ruhm alleine reicht nicht Wie der gefeierte Entdecker Albert Neisser (1855–1916) in Leipzig zu akademischen Weihen in der Dermatologie kam. Hautarzt.

[40] Hüntelmann AC (2011). Paul Ehrlich. Leben, Forschung, Ökonomien, Netzwerke.

[41] Neisser A (1898). Was wissen wir von einer Serumtherapie bei Syphilis und was haben wir von ihr zu erhoffen? Eine kritische Übersicht und Materialiensammlung Festschrift gewidmet Filipp Josef Pick. II Teil. Arch Dermatol Syph.

[42] Quidde (1900). Arme Leute in Krankenhäusern.

[43] Reuland A (2004) Menschenversuche in der Weimarer Republik. Universitätsbibliothek Heidelberg, Heidelberg S 11–20. https://books.ub.uni-heidelberg.de/heibooks/reader/download/260/260-4-77697-2-10-20170523.pdf. Zugegriffen: 2. Jan. 2023

[44] Pagel L (1900). Zum Fall Neisser. Dtsch Medizinalz.

[45] Pagel L (1905) Über den Versuch am lebenden Menschen. Dtsch Aerzte Z 7(9, 10):193–198, 217–228, 226

[46] Sabisch K (2007). Das Weib als Versuchsperson Medizinische Menschenexperimente im 19. Jahrhundert am Beispiel der Syphilisforschung.

[47] Wagner E (2011). Der Arzt und seine Kritiker. Zum Strukturwandel medizinkritischer Öffentlichkeiten am Beispiel klinischer Ethik-Komitees.

[48] Krischel M, Nebe J (2022). Forschungs- und Publikationsethik in der Zahnmedizin. „Publish or perish“ – zwischen Promotion und Plagiat. Zahnärztl Mitt.

[49] Buschke A (1916). Albert Neisser. Derma Wochenschr.

[50] Jadassohn J (1916). Albert Neisser.

[51] Moll F, Krischel M, Fangerau H, American Urological Association (2012). Albert Neisser and the first prussian directive on informed consent. Skeletons in the closet indignities and injustices in medicine.

[52] Ministerium der Geistlichen, Unterrichts- und Medizinalangelegenheiten (1901). Anweisung an die Vorsteher der Kliniken, Polikliniken und sonstigen Krankenanstalten.

[53] Vollmann J (2000). Aufklärung und Einwilligung in der Psychiatrie Ein Beitrag zur Ethik in der Medizin.

[54] Werner P (2002). Der Heiler. Tuberkuloseforscher Friedrich F. Friedmann.

[55] Sass HM (1983). Reichsrundschreiben 1931: Pre-Nuremberg German regulations concerning new therapy and human experimentation. J. Med. Philos..

[56] Saretzki Th (2000). Reichsgesundheitsrat und Preußischer Landesgesundheitsrat in der Weimarer Republik.

[57] Vollmann J, Winau R (1996). Informed consent in human experimentation before the Nuremberg Code. Br Med J.

[58] Jonas H 2017 Das Lübecker Impfunglück von 1930in der Wahrnehmung von Zeitzeuginnen und Zeitzeugen. Diss Med, Lübeck.

[59] Annas GJ (1992). The changing landscape of human experimentation: Nuremberg, Helsinki und beyond. Health Matrix J Law Med.

[60] Deklaration von Helsinki. https://www.bundesaerztekammer.de/fileadmin/user_upload/downloads/pdf-Ordner/International/Deklaration_von_Helsinki_2013_20190905.pdf. Zugegriffen: 31. Apr. 2021

[61] Roelcke V (2017). Informed Consent and Social Vulnerability in Human Subject Research: the German Richtlinien/guidelines for Human Subject research, ca 1931–1961/64. World Med J.

[62] Weindling P (2017). From clinic to concentration camp: reassessing Nazi medical and racial research, 1933–1945.

[63] Winau R, Vollmann J (1996). Informed consent in human experimentation before the Nuremberg code. Br Med J.

[64] Groß D, Lenk C, Duttge G, Fangerau H (2014). Nürnberger Kodex‘. Handbuch Ethik und Recht der Forschung am Menschen.

[65] Roelcke V (2009). Die Sulfonamid-Experimente in nationalsozialistischen Konzentrationslagern: Eine kritische Neubewertung der epistemologischen und ethischen Dimension. Med Hist J.

[66] Weindling P (2004). Nazi medicine and the Nuremberg trials: from medical war crimes to informed consent.

[67] Atzor S, Gokhale S, Doherty M (2013). Will the EU clinical trials regulation support the innovative industry in bringing new medicines faster to patients?. Pharm Med.

[68] Fangerau H, Lenk Chr, Duttke G, Fangerau H (2014). Geschichte der Forschung am Menschen. Handbuch Ethik und Recht der Forschung am Menschen.

[69] Fangerau H, Schulz S, Steigleder K, Fangerau H, Paul N (2006). Ethik der medizinischen Forschung. Geschichte, Theorie und Ethik der Medizin. Eine Einführung.

[70] Ziegler T M 2014 Das Humanexperiment in der medizinischen Forschung in der Diskussion der verfassten Ärzteschaft der Bundesrepublik Deutschland im Spiegel des Deutschen Ärzteblatts von 1949–1978 Diss Med Tübingen.

[71] Moll F, Rathert P (1999). The surgeon and his intention: Gustav Simon (1824–1876), his first planned nephrectomy and further contributions to urology. World J Urol.

[72] Moll F, Frank M, Moll F (2006). Die Blase des Theodor Baum. Am Anfang war Napoleon. Kölner Krankenhausgeschichten.

[73] Maehle AH (2003). Ärztlicher Eingriff und Körperverletzung Zu den historisch-rechtlichen Wurzeln des Informed Consent in der Chirurgie 1892–1940. Würzb Med Hist Mitt.

[74] Reitsma AM, Moreno JD (2002). Ethical Ethical regulations for innovative surgery: the last frontier?. J Am Coll Surg.

[75] Winau R, Wiesemann C, Frewer A (1996). Medizin und Menschenversuch. Zur Geschichte des „Informed Consent“. Medizin und Ethik im Zeichen von Auschwitz. 50 Jahre Nürnberger Ärzteprozess.

[76] Huber-Lang M, Gebhard F (2014) Klinische Prüfung chirurgischer Eingriffe. In: Lenk Chr, Duttke G, Fangerau H (Hrsg) Handbuch Ethik und Recht der Forschung am Menschen. Springer, Berlin, S 17–24 10.1007/978-3-642-35099-3

[77] Simon A, Marckmann G (2015). Patientenautonomie und informed consent. Praxisbuch Ethik in der Medizin.

[78] Maehle AH, Paul N, Schlich Th (1998). Werte und Normen: Ethik in der Medizingeschichte. Medizingeschichte: Aufgaben, Probleme, Perspektiven.

[79] Jütte R (2017). Arzt und Ethos Aufklärung und „informed consent“. Dtsch Arztebl.

[80] Marckmann G, Sandberger G, Wiesing U (2010). Begrenzung lebenserhaltender Behandlungsmaßnahmen: Eine Handreichung für die Praxis auf der Grundlage der aktuellen Gesetzgebung. Dtsch Med Wochenschr.

[81] Marckmann G, Michalsen A, Michalsen A, Hartog C (2013). Entscheidungsfindung zur Therapiebegrenzung. End-of-Life Care in der Intensivmedizin.

[82] Corvinius U (2007) Medizinische Forschung mit nichteinwilligungsfähigen erwachsenen Patienten Literaturrecherche und Statuserhebung zum Umgang mit Aufklärung, Einwilligung und klinischer Datenverarbeitung. Diss Med. Univ Gießen. https://geb.uni-giessen.de/geb/volltexte/2008/5280/pdf/CorvinusUrsula-2007-12-03.pdf. Zugegriffen: 7. Juni 2021

[83] Hick C (2007). Klinische Ethik.

[84] Fangerau H, Noack T, Fangerau H, Vögele J (2007). Ethik eine Einführung. Geschichte, Theorie und Ethik der Medizin.

[85] Marckmann G (2022). Praxisbuch Ethik in der Medizin.

[86] Heinrichs B (2017) Forschung am Menschen. https://www.bpb.de/themen/umwelt/bioethik/257445/forschung-am-menschen/. Zugegriffen: 7. Jan. 2023

[87] Münchener Freie. Presse. 30.10.1898, Nr. 247 „Arme Leute“ S. 29–31

